# Editorial: Model systems to study the mechanisms of neural development and disease

**DOI:** 10.3389/fnmol.2022.1006888

**Published:** 2022-08-31

**Authors:** N. Sumru Bayin, Paula Alexandre, Parthiv Haldipur

**Affiliations:** ^1^Wellcome Trust/Cancer Research UK Gurdon Institute, Cambridge University, Cambridge, United Kingdom; ^2^University College London Great Ormond Street Institute of Child Health, London, United Kingdom; ^3^Center for Integrative Brain Research, Seattle Children's Research Institute, Seattle, WA, United States

**Keywords:** brain, disease, animal models, development, cellular models

Experimental models ranging from vertebrate and invertebrate models to *in vitro* cellular systems have been used to study the mechanisms underlying brain development and disease. Each of these models has specific strengths and caveats in recapitulating human biology. Some make use of elaborate genetic manipulations and are suitable for longitudinal studies while others may be more advantageous for large scale experiments. The need of the hour is to compare the differences and commonalties of findings obtained from different model systems and identify the benefits and drawbacks of each model system.

Human developmental studies of the brain have relied heavily on “macro scale” imaging, and pathology which provide discontinuous snapshots of the developing brain that omit evidence for what the brain may have looked like before or what it would eventually develop into. These approaches do not inform on the molecular and cellular mechanisms operational during brain development.

*In vitro* cellular systems and animal models have an advantage in this regard since they provide the luxury of performing sophisticated and reproducible experimentation which can help us decipher cellular and molecular mechanisms more easily and follow the development and emergence of phenotypes over time. The use of experimental models is also based on the principle that at least some features of human brain development are conserved across vertebrate and invertebrate species and can be recapitulated *in vitro*. In addition, genetically modified animal models what have been used to perform structure-function analyses, *in vivo* imaging and longitudinal studies to follow phenotypes over time, can help elucidate gene function, and vastly improve our understanding of basic neurobiological processes such as neuronal specification, neuronal circuit formation and function. These models also aid in the development of targeted therapies against neurodevelopmental disorders. However, the jury is still out on the extent to which animal and cellular models truly and fully recapitulate human brain development and associated pathologies.

Recent studies on the human brain that have utilized imaging, histology and other high throughput approaches such as single cell genomics that are aimed at unraveling the molecular complexity of the human brain, have highlighted that many animal models, including rodents do not fully recapitulate human brain development, particularly cerebral and cerebellar development. This is likely due to the large evolutionary expansion of the human brain both in size and complexity to fulfill higher-order functions. For example, the human brain has expanded progenitor zones and cell types that are either absent or reduced significantly in rodents, and even non-human primates. Such findings highlight the necessity of humanized animal and cellular models as well as comparative analyses. Identification of human-specific developmental mechanisms has resulted in a renewed focus on human tissue-based studies which have helped characterize these foundational differences. However, human tissue is rare and not readily available to most labs. A paucity of standardized protocols for sophisticated experimentation, live imaging and human slice cultures are some of the present challenges for human studies. *In vitro* cellular models like brain organoids have also been useful in understanding human diseases, particularly of the cerebral cortex since cerebral organoids have been shown to mimic many early aspects of human cerebral development. However, these models fail to fully recapitulate the later stages of neural development and consume a lot of resources. Additionally, human tissue, particularly from malformed brains is scarce and not readily available to most. Finally, challenges of performing elaborate genetics and whole organ/live imaging approaches present complications for human studies and humanized models. Collectively all these highlight the value and benefit of utilizing multiple models and different expertise when investigating neural development and disease, both for practical and evo-devo angles.

In this Research Topic, our goal is to introduce the functionality of various *in vitro* and *in vivo* models and showcase their effectiveness in improving our understanding of brain development and disease, in order to facilitate a cross model system discussion on neural development and disease mechanisms. Here, we present valuable review articles that discuss brain tumors (Antonica et al.), aging (Chaudhar et al.) and genetic disorders affecting brain development (Biel et al.) from the perspective of the disease models; modeling neural polarity mechanisms and their role in developmental disease (Solecki), and the power of imaging approaches and emerging non-mammalian models in neuroscience (Haynes et al.). In addition to these resources, we have research articles that describe the use of models to identify the molecular mechanisms of various aspects of neurodevelopmental aging from central and peripheral nervous system development (Delalande et al.; Jose et al.; Natarajan et al.), and associated pathophysiology (Roy et al.; Kelani et al.).

## Author contributions

All authors listed have made a substantial, direct, and intellectual contribution to the work and approved it for publication.

## Funding

This work was supported by Brain and Behavior Research Foundation Young Investigator Award and NIH-R21 NS117848 to PH, NIH/NINDS (K99/R00 NS112605-01) and core support from Wellcome Trust (#092096) to NS, and Wellcome Human Developmental Biology Initiative (#182625) to PA.

## Conflict of interest

The authors declare that the research was conducted in the absence of any commercial or financial relationships that could be construed as a potential conflict of interest.

## Publisher's note

All claims expressed in this article are solely those of the authors and do not necessarily represent those of their affiliated organizations, or those of the publisher, the editors and the reviewers. Any product that may be evaluated in this article, or claim that may be made by its manufacturer, is not guaranteed or endorsed by the publisher.

